# Suffocation Injuries in the United States: Patient Characteristics and Factors Associated with Mortality

**DOI:** 10.5811/westjem.2018.4.37198

**Published:** 2018-06-04

**Authors:** Roula Sasso, Rana Bachir, Mazen El Sayed

**Affiliations:** * American University of Beirut Medical Center, Department of Emergency Medicine, Beirut, Lebanon; † American University of Beirut Medical Center, Department of Emergency Medicine, Emergency Medical Services and Prehospital Care Program, Beirut, Lebanon

## Abstract

**Introduction:**

Asphyxiation or suffocation injuries can result in multi-organ damage and are a major cause of morbidity and mortality among different age groups. This study aims to describe characteristics of patients presenting with suffocation injuries to emergency departments (EDs) in the United States (U.S.) and to identify factors associated with mortality in this population.

**Methods:**

We conducted a retrospective cross-sectional study using the 2013 U.S National Emergency Department Sample database. ED visits with primary diagnoses of intentional or accidental suffocation injury, and injury by inhalation and aspiration of foreign bodies or food (ICD-9-CM codes) were included. We performed descriptive statistics to describe the study population. This was followed by multivariate analyses to identify factors associated with mortality.

**Results:**

We included a total of 27,381 ED visits for suffocation injuries. Most suffered from either inhalation and ingestion of food causing obstruction of respiratory tract or suffocation (51.6%), or suicide and self-inflicted injury by hanging, strangulation, and suffocation (39.4%). Overall mortality was 10.9%. Over half (54.7%) of the patients were between 19 and 65 years old. Males were more common than females (59.1% vs. 40.9%). Over half of the patients (54.9%) were treated and released from the ED. Factors associated with increased mortality included male gender, young age (4–18 years), diseases of the cardiac, respiratory, genitourinary and neurologic systems, intentional self-harm, and self-payer status.

**Conclusion:**

Mortality from suffocation injuries remains high with significant burden on children and adolescents and on patients with intentional injuries. Tailored initiatives targeting identified modifiable factors through implementation of behavioral and environmental change can reduce the risk of suffocation injury and improve clinical outcomes of affected victims.

## INTRODUCTION

Asphyxiation or suffocation can be defined as the deprivation of oxygen supply to body tissues and can result from mechanical or non-mechanical constriction of the airway or from a decrease in breathable gas in the respired surrounding atmosphere.^1^ Suffocation and asphyxiation can vary at the forensic pathology level;^1^ however, both can be used interchangeably to report a decrease in oxygen delivery to the lungs resulting in deprivation of oxygen or hypoxia.^2^, ^3^

Suffocation injuries and death can result from suicidal attempt, assault or accidental injury. In parts of Europe and Asia, intentional asphyxiation by hanging is the leading manner of suicide attempts.^4^,^5^ In the United States (U.S.), the rate of suicide by intentional asphyxiation is second only to suicide by firearms.^6^ Choking is a form of unintentional asphyxiation: choking was the third leading cause of unintentional deaths in the U.S. between 2000 and 2013 in adults aged 65 years or older^7^ and a leading cause of morbidity and mortality among children aged less than 3 years.^8^ In addition to death and multi-organ damage, complications of asphyxiation include cardiopulmonary injuries and neurological injuries, in addition to orthopedic injuries with hangings and strangulation.^9^–^11^

The current medical literature describing suffocation injuries and clinical outcomes is limited and is mostly focused on death from asphyxiation by hanging or strangulation injuries.^12^–^16^ Available medical literature suggests that cardiopulmonary resuscitation at the scene of suffocation injury is associated with improved clinical outcomes while longer duration of hanging is associated with increased mortality in cases of hanging asphyxiation.^17^ Low Glasgow Coma Scale on arrival to the emergency department (ED) has also been associated with poor clinical outcomes.^18^ Understanding the epidemiology of suffocation injuries, characteristics of affected victims and factors associated with mortality is important for physicians and policymakers to tailor prevention initiatives and mitigation strategies.

Our goal was to describe the characteristics of patients presenting with suffocation injuries to EDs in the U.S and to identify factors associated with mortality in this population.

## METHODS

### Study Design

This retrospective cross-sectional study used the 2013 public release U.S National Emergency Department Sample (NEDS). NEDS is the largest all-payer (ED) database available in the U.S. and is part of the Healthcare Utilization Project (HCUP), which is supported by the Agency of Healthcare and Research Quality.^19^ The NEDS database contains data from approximately 30 million ED visits each year.^20^ In 2013, the NEDS database collected data for 134,869,015 ED visits from 947 hospitals across 30 states, representing an approximate 20% stratified sample of U.S. hospital-based EDs. The NEDS dataset is released three years after its collection.

All members of the research team who were involved in using the NEDS database completed the HCUP data use agreement training course and signed the Nationwide Data Use Agreement. The institutional review board (IRB) at the American University of Beirut provided IRB exemption for the use of the NEDS public release dataset.

We identified ED visits for patients with suffocation injury using diagnosis codes (*International Classification of Disease - 9 - Clinical Modification* [ICD-9-CM]) listed in Table 1. These encompassed injuries by accidental mechanical suffocation, intentional and unintentional injuries by hanging, strangulation and suffocation, injury by inhalation and aspiration of foreign bodies or food.

**Population Health Research Capsule**

What do we already know about this issue?

Suffocation injuries result in multi-organ damage and are a major cause of morbidity and mortality among different age groups.

What was the research question?

What are the characteristics of patients with suffocation injuries and factors associated with mortality in the United States?

What was the major finding of the study?

Mortality from suffocation is high with a significant burden on younger individuals and those with intentional injuries

How does this improve population health?

Familiarity with patients at risk of suffocation injuries can improve clinical outcomes and allow for implementation of initiatives that target behavior changes in this population.

Variables available from the NEDS database included patient characteristics and comorbidities, type of injury and injury intent, patient disposition, admission rates, hospital length of stay and cost. Clinical outcome was defined as mortality in ED or during hospital stay (yes/no).

### Statistical Analysis

We performed statistical analysis with SPSS (version 24) statistic software package. The description of the sociodemographic, clinical and administrative characteristics was presented as frequencies, percentages, and 95% confidence interval (CI) for the categorical variables and mean and 95% CI for the continuous variables. We used the Rao-Scott chi-square test for complex sample design to determine the significance of the statistical association between the independent variables and mortality (yes/no), the dependent variable. All variables that were found to be statistically significant in the bivariate level were included in a logistic regression model to determine the factors significantly associated with mortality. We presented results of the multivariate analysis as odds ratio (OR) along with the corresponding 95% CI. Convenient methods including CSDESCRIPTIVES, CSTABULATE, and CSLOGISTIC for complex survey design were performed to calculate accurate estimates. A value was considered statistically significant at a p-value less than or equal to 0.05.

## RESULTS

We included a total of 27,381 ED visits for suffocation injuries in the study. The resulting incidence for suffocation injuries in 2013 in the U.S. was 20 per 100,000 ED visits. Inhalation and ingestion of food causing obstruction of respiratory tract or suffocation was the most common presentation (51.6%), followed by suicide and self-inflicted injury by hanging, strangulation, and suffocation (39.3%) (Table 1).

Over half of the patients (54.7%, 95% CI [53.5 – 55.9]) were between 19–65 years. Males (59.1%, 95% CI [57.9 – 60.2]) were more common than females, and most patients had chronic conditions (72.8%, 95% CI [71.8 – 73.9]). The most common body system indicators (defined as a collective designation of body system specific ICD-9-CM codes) were injury and poisoning (80.2%, 95% CI [79.2 – 81.1]) and mental disorders (47.4%, 95% CI [46.2 – 48.5]). The majority of patients had reported injuries on presentation (80.3%, 95% CI [79.4 – 81.2]) and the most common reported method of injury was injury by assault (3.4%, 95% CI [3.0–3.9]) followed by injury by poisoning (2.0%, 95% CI [1.7–2.3]). Patients had mainly minor injuries (Injury Severity Score <15) (99.5%, 95% CI [99.3 – 99.7]). Intentional self-harm was recorded in 40.1% (95% CI [38.9 – 41.2]) of cases with injuries. ED suffocation related visits were similar across all seasons. Most visits (71.9%, 95% CI [70.8 – 72.9]) were during weekdays and Medicare was the most common type of coverage (29.3%, 95% CI [28.3 – 30.0]) (Table 2).

Most patients were either treated and released from the ED (54.9%, 95% CI [53.8 – 56.1]) or admitted to the same hospital as presentation (33.6%, 95% CI [32.6 – 34.7]). Patients who were admitted had an average length of stay of 6.2 days (95% CI [5.8– 6.7]). Overall mortality in the study population was 10.9% (95% CI [10.2 – 11.7]). Mortality rates ranged from 9.6% (95% CI [8.6 – 10.8]) for patients with “suicide and self-inflicted injury by hanging strangulation and suffocation” to 30.7% (95% CI [22.5 – 40.4]) for patients with “hanging, strangulation, or suffocation, undetermined whether accidentally or purposely inflicted.” Those with accidental mechanical suffocation had a mortality rate of 11% (95% CI [10.0–12.0]). The mean for total ED charges was $3,620.20 (95% CI [3531.6 – 3708.7]) (Table 3).

We performed a bivariate analysis (not shown) to compare patients’ characteristics by outcome (mortality); significant differences were noted between the two groups. Patients who died were more likely to be older, have chronic conditions and be of male gender. They also had more admissions during weekends and had more injuries reported. Higher frequencies of mental health disorders, of intentional self-harm, injury by poisoning and injury by assault were, however, noted in the group of patients who survived. There was no difference in patient outcomes by season of admission or by injury severity.

In the multivariate analysis, factors that were significantly associated with increased mortality after suffocation (Table 4) included male gender (OR [1.3], 95% CI [1.1–1.6]), disease of the circulatory system (OR [11.6], 95% CI [8.9–15.1]), diseases of the nervous system (OR [3.0], 95% CI [2.4–3.8]), diseases of the respiratory system (OR[1.9], 95% CI [1.6–2.4]) and diseases of the genitourinary system (OR [1.5], 95% CI [1.1–1.9]). Additional factors that were also associated with increased mortality included age category 4–18 years (OR [1.8], 95% CI [1.2–2.7]), intentional self-harm (OR [2.0], 95% CI [1.5–2.7]) and having one or no injury reported (OR [1.5], 95% CI [1.1–2.0]). Mental health disorders (OR [0.4], 95% CI [0.3–0.5]) were found to be negatively associated with mortality.

## DISCUSSION

This study examined suffocation injuries in a large national sample of ED visits; it is the largest study to date to report on this medical condition and to attempt to identify its burden using a national sample in the U.S. While the mechanisms of injury resulting in suffocation and asphyxiation are numerous, several forms of asphyxiation are uncommon and under-reported. This is evident in the medical literature with several forms of asphyxiation described only in case reports or case series.^21^, ^22^.Some studies have reported mortality rates in more common forms of suffocation injuries such as hanging and strangulation.^10^,^17^,^18^,^23^,^24^ Our study, however, addressed all forms of suffocation injuries using a national sample, in an attempt to identify the burden of this disease and avoid overlooking under-reported and uncommon forms of injury that result in suffocation and asphyxiation.

The incidence of suffocation injury in 2013 among ED patients was 20 per 100,000 individuals, and the mortality rate was 10.9% (95%CI [10.1–11.7]) with varying mortality rates, ranging from 9.6% (suicide and self-inflicted injury by hanging, strangulation, and suffocation) to 30.7% (undetermined if self-inflicted or accidental). A study in Japan showed a 77% mortality rate in individuals presenting with suicidal-intent hanging injury,^17^ whereas another study in Australia showed a mortality rate of 12% for the same form of injury.^18^ This discrepancy in mortality rates could be attributed to different factors including lack of standardized management of suffocation injuries,^25^–^27^ higher injury acuity, or different groups of patients included in other studies.

Additionally, 47.4% of patients in this study had a mental health condition and 40.1% had intentional injury reported. These results are in line with previous studies suggesting that suffocation injuries are commonly a result of intentional self-harm or suicidal attempt and are likely to occur in patients with a history of psychiatric disorders.^23^ In fact, a previous study examining deaths in adolescents due to hanging injury revealed that the majority of hangings (98.4%) were the result of a suicidal attempt.^28^ Similarly, deaths due to plastic-bag suffocation were mostly in adults and resulted from suicidal attempts.^22^ Our results also showed that intentional self-harm was associated with higher odds of mortality after controlling for confounding factors in the multivariate analysis. While the literature assessing the impact of intentional injury on mortality in patients with suffocation injuries is scarce, studies exploring this impact in injury/trauma patients in general have reported higher mortality associated with intentional injury.^30^, ^31^

Males were both more likely to present with and die from suffocation injuries. The available published literature reports conflicting data on the gender role in suffocation injuries by hanging.^12^,^17^,^18^,^23^,^26^ by inhalation of helium gas,^6^,^32^, by plastic-bag suffocation,^22^ and by autoerotic asphyxiation.^21^ The various mechanisms of suffocation may have contributed to this inconsistency in impact of gender on outcomes after suffocation. Other confounding factors such as intentional injury may also explain this inconsistency. Several studies have suggested that suffocation related to suicidal intent is more common in males,^9^,^32^,^33^–^35^ while suffocation related to assault or homicidal intent is more likely to occur in females.^5^,^36^,^37^.

Patients aged 4–18 years were observed to have higher odds of mortality compared to other age groups. The existing literature does not provide clear evidence for this. However, some studies suggest that children are more vulnerable to have complete airway obstruction and are more prone to delayed airway edema after strangulation, due to the relatively small size of the airway.^38^, ^39^ Additionally, we can speculate that children are more likely than adults to experience prolonged unintentional suffocation as they are often left unattended and incapable of self-help. More research investigating the relationship between age and mortality among patients with suffocation injuries could help develop age-specific prevention strategies.

This study also showed that individuals with disease of the circulatory system, nervous system, respiratory system and genitourinary systems are significantly more likely to die from suffocation injuries. This was expected since patients with baseline cardiac, respiratory and kidney diseases are more likely to have poorer clinical outcomes.^40^–^42^ Mental disorders seemed to be negatively associated with mortality in patients presenting with suffocation injuries. While mental disorders are associated with higher natural and unnatural cause mortality,^43^ some studies have demonstrated mental disorders to be protective in trauma patients.^44^,^45^ Individual with suffocation injuries who survive are more likely to undergo psychiatric disease evaluation and to get diagnosed in the ED and in hospital with a psychiatric disorder, which may result in a higher frequency of mental disorders in surviving patients. Additionally, patients could suffer from psychiatric disorders that are a result of the traumatic injury experienced. However, further studies are needed to evaluate the effect of pre-existing mental disorders on patients with suffocation injuries.

## LIMITATIONS

This study has limitations inherent to its retrospective nature. NEDS is, however, a large U.S. national database of ED visits and the study results can be generalized to other patients presenting with suffocation injuries in the U.S. or in other developed settings. The data was obtained from the NEDS database using ICD-9-CM codes for suffocation diagnosis. There could be an underestimation of the actual suffocation injury rates due to variations in coding in the 947 hospitals included in the database. It is also possible that many patients who died as a result of suffocation might not have been transported to the ED and therefore were not included in this study, which could potentially have led to underestimation of mortality rate of asphyxiation. The NEDS database is de-identified, so we could not identify patients with suffocation injury readmissions. Considering that a high percentage of patients had mental disorders or a history of injury and poisoning, it is likely that the number of readmissions in our selected population is significant. Other studies excluding readmissions might identify other factors associated with mortality in patients with suffocation injury. Patients presenting with a recurrent suffocation injury are also more likely to have poorer prognosis than those presenting with a suffocation injury for the first time.

This study included patients with suffocation from different mechanisms. Even though mortality rates were reported for different mechanisms when possible, restrictions related to availability of clinical variables limited our ability to draw more specific recommendations about clinical management or to identify whether the associated clinical conditions were co-morbid conditions or arose as a result of the asphyxiation.

## CONCLUSION

Mortality from suffocation injuries remains high in the U.S with a significant burden on children and adolescents and on patients with intentional injuries. Familiarity with the characteristics of patients at risk of suffocation injuries and with factors associated with increased mortality after such injuries is important to help improve clinical outcomes. Additionally, tailored initiatives implementing behavior and environment change and targeting populations at risk of suffocation injuries are needed.

## Figures and Tables

**Table 1:** International Classification of Disease - 9 - Clinical Modification (ICD-9-CM) codes with injury description.

Injury description	ICD-9 CM codes	N (%)
Accidental mechanical suffocation	E913.0, E913.1, E913.2, E913.3, E913.8, E913.9	602 (2.2)
Suicide and self-inflicted injury by hanging, strangulation, and suffocation	E953.0, E953.1, E953.8, E953.9	10,765 (39.3)
Hanging, strangulation, or suffocation, undetermined whether accidentally or purposely inflicted	E983.0, E983.1, E983.8, E983.9	513 (1.9)
Inhalation and ingestion of food causing obstruction of respiratory tract or suffocation	E911	14,140 (51.6)
Asphyxiation and strangulation	994.7	4,565 (16.7)

**Table 2:** Study characteristics of patients with suffocation injuries.

Characteristics	Frequency (N=27381)	Percentage (95% CI)
Gender		
Male	16173	59.1 (57.9 – 60.2)
Female	11209	40.9 (39.8 – 42.1)
Age		
Newborn -3	2057	7.5 (6.9 – 8.1)
4 –18	3892	14.2 (13.4 – 15.1)
19 – 65	14972	54.7 (53.5 – 55.9)
≥66	6446	23.6 (22.6 – 24.6)
Chronic conditions		
Chronic conditions	19943	72.8 (71.8 – 73.9)
No chronic conditions	7438	27.2 (26.1 – 28.2)
Body system indicator^1^		
Injury and poisoning	21951	80.2 (79.2 – 81.1)
Mental disorders	12968	47.4 (46.2 – 48.5)
Factors influencing health status and contact with health services^2^	9659	35.3 (34.2 – 36.4)
Symptoms, signs, and ill-defined conditions	9298	34.0 (32.9 – 35.1)
Diseases of the circulatory system	9007	32.9 (31.8 – 34.0)
Injury diagnosis reported on record		
Injury reported	21995	80.3 (79.4 – 81.2)
No injury diagnosis reported	5386	19.7 (18.8 – 20.6)
Method of injury^3^		
Injury by assault	940	3.4 (3.0–3.9)
Injury by poisoning	535	2.0 (1.7–2.3)
Injury by falling	328	1.2 (1.0–1.5)
Injury by cutting or piercing	309	1.1 (0.9–1.4)
Injury from being struck	273	1.0 (0.8–1.3)
More than one injury diagnosis reported on record		
One or no injury diagnosis reported	23728	86.7 (85.8 – 87.4)
More than one injury diagnosis reported	3654	13.3 (12.6 – 14.2)
Injury severity score		
Minor trauma (0–15)	27239	99.5 (99.3 – 99.7)
Major trauma (16–75)	130	0.5 (0.3–0.7)
Intentional self-harm indicated on record		
Intended self-harm	10967	40.1 (38.9 – 41.2)
No intended self-harm	16414	59.9 (58.8 – 61.1)
Season of admission		
Summer	6325	26.4 (25.2–27.5)
Spring	6188	25.8 (24.7–26.9)
Autumn	6073	25.3 (24.2–26.4)
Winter	5411	22.5 (21.5–23.6)
Admission day		
Monday – Friday	19675	71.9 (70.8 – 72.9)
Saturday - Sunday	7706	28.1 (27.1 – 29.2)
Urban-rural location of patient residence		
Large central metropolitan	6721	24.8 (24.1 – 25.5)
Medium metropolitan	6196	22.8 (22.2 – 23.5)
Large fringe metropolitan	5718	21.1 (20.4 – 21.8)
Micropolitan	3271	12.1 (11.6 – 12.5)
Small metropolitan	2871	10.6 (10.0 – 11.2)
Not metropolitan or micropolitan	2356	8.7 (8.3 – 9.1)
Primary expected payer		
Medicare	7991	29.3 (28.3 – 30.4)
Medicaid	6023	22.1 (21.1 – 23.1)
Private including Health Maintenance Organization (HMO)	7225	26.5 (25.5 – 27.6)
Self-payer	3836	14.1 (13.3 – 14.9)
No charge	242	0.9 (0.7 – 1.1)
Other	1916	7.0 (6.4 – 7.7)
Median household income		
$1 – $37,999	7417	28.0 (27.0 – 29.0)
$38,000 – $47,999	7496	28.3 (27.3 – 29.4)
$48,000 – $63,999	6582	24.9 (23.9 – 25.9)
$64,000 or more	4987	18.8 (18.0 – 19.7)

*CI*, confidence interval.

1 Only the five most common body system indicators are shown. Other body system indicators listed from most to least frequent are as follows: (1) Disease of the respiratory system; (2) endocrine, nutritional and metabolic disease and immunity disorders; (3) diseases of the nervous system and sense organs, (4) disease of the digestive system; (5) diseases of the musculoskeletal system; (6) diseases of the genitourinary system; (7) disease of the blood and blood-forming organs; (8) infectious and parasitic disease; (9) diseases of the skin and subcutaneous tissue; (10) neoplasms; (11)congenital anomalies; (12) certain conditions originating in the perinatal period; and (13) complications of pregnancy, childbirth and the perineum.

2 Includes (1) newborns and infants; (2) vaccinations and inoculations; (3) suspected exposure to communicable diseases; (4) patients who are either a carrier of a disease or have the sequelae or residual of a past disease or condition; (5) patient’s past medical condition that no longer exists and is not receiving any treatment, but that has the potential for recurrence or patients with family member(s) who has had a particular disease that causes the patient to be at higher risk of also contracting the disease; (6) screening encounter;, (7) observational encounters; (8) aftercare encounters; (9) follow-up encounters; (10) donors; (11) counseling encounters,;(12) encounters for obstetrical and reproductive services; (13) routine and administrative examinations; and (14) miscellaneous encounters.

3 Only the five most common Injury methods are shown: Other Injury methods listed from most to least frequent are as follows: (1) injury involving motor vehicle traffic; (2) injury involving natural or environmental causes; and (3) injury by fire, flame, or hot objects.

**Table 3:** Outcomes after suffocation injuries.

Outcomes – categorical variables	Frequency (N=27381)	Percentage (95% CI)
Disposition from the ED		
ED visit in which the patient is treated and released	15041	54.9 (53.8 – 56.1)
ED visit in which the patient is admitted to the same hospital	9210	33.6 (32.6 – 34.7)
ED visit in which the patient is transferred to another short-term hospital	1941	7.1 (6.5 – 7.7)
ED visit in which the patient died in the ED	1076	3.9 (3.5 – 4.4)
ED visits in which patient was not admitted, destination unknown	112	0. 4 (0.3 – 0.6)
Death		
Death in ED/hospital	2976	10.9 (10.2 – 11.7)
No death	24280	89.1 (88.3 – 89.8)

Outcomes-continuous variables	N	Mean (95% CI)
Total charge for ED ($)	27358	3620.2 (3531.6 – 3708.7)
Length of stay (days)	9210	6.2 (5.8 – 6.6)

*CI,* confidence interval, *ED,* emergency department.

**Table 4:** Factors associated with mortality after suffocation injury.

Variables	Odds ratio	95% CI	P value
Gender			
Female	1.0	-	-
Male	1.3	1.1–1.6	0.008
Age (years)			
≥66	1.0	-	-
19 – 65	1.2	0.9–1.6	0.256
4 –18	2.2	1.4–3.4	0.001
0 – 3	0.9	0.5–1.8	0.912
Admission day			
Weekend	1.0	-	-
Weekday	0.8	0.7–1.0	0.078
Body system indicators			
Diseases of the circulatory system (No)	1.0	-	-
Yes	11.6	8.8–15.1	<0.001
Diseases of the nervous system and sense organs (No)	1.0	-	-
Yes	3.0	2. 5–3.8	<0.001
Diseases of the respiratory system (No)	1.0	-	-
Yes	1.9	1.6–2.4	<0.001
Diseases of the genitourinary system (No)	1.0	-	-
Yes	1.5	1.1–1.9	0.003
Factors influencing health status and contact with health services (No)	1.0	-	-
Yes	0.8	0.7–0.9	0.010
Diseases of the digestive system (No)	1.0	-	-
Yes	0.6	0.4–0.7	<0.001
Diseases of the musculoskeletal system (No)	1.0	-	-
Yes	0.4	0.3–0.6	<0.001
Mental disorders (No)	1.0	-	-
Yes	0.4	0.3–0.5	<0.001
Injury and poisoning (No)	1.0	-	-
Yes	1.1	0.6–2.0	0.739
Intentional self-harm			
Unintentional self-harm	1.0	-	-
Intentional self-harm	2.1	1.5–2.7	<0.001
More than one injury diagnosis			
More than one injury diagnosis	1.0	-	-
One or no injury diagnosis	1.5	1.1–2.0	0.011
Injury diagnosis reported on record			
No injury diagnosis reported	1.0	-	-
Injury reported	1.5	0.8–2.7	0.180
Method of Injury			
Injury by falling (No)	1.0	-	-
Yes	1.3	0.7–2.6	0.446
Injury by poisoning (No)	1.0	-	-
Yes	0.4	0.1–1.3	0.124
Injury by assault (No)	1.0	-	-
Yes	1.1	0.6–1.8	0.912
Primary expected payer			
Self-payer	1.0	-	-
Medicare	0.4	0.3 – 0.6	<0.001
Medicaid	0.3	0.2–0.4	<0.001
Private including Health Maintenance Organization (HMO)	0.5	0.3 – 0.6	<0.001
No charge	0.4	0.2 – 1.0	0.063
Other	0.5	0.4 – 0.8	0.003

*CI,* confidence interval.
